# Supplementary material for: Sperm-Associated Antigen 5 Knockout Reduces Doxorubicin and Docetaxel Resistance in Triple-Negative Breast Cancer MDA-MB-231 and BT549 Cells
Source: Cancers (Basel). 2024 Mar 24;16(7):1269. doi: 10.3390/cancers16071269 (PMC11010853; doi:10.3390/cancers16071269)
Supplement: Supplementary file 1 [file cancers-16-01269-s001.zip › cancers-2909491-supplementary.pdf]

Supplementary Materials

To screen the isolated knockout clones, a forward primer Puro5-fwd and a reverse primer SPAG5R3-rev targeting the sequence of the puromycin-resistant gene in donor plasmid and the *SPAG5* gene genomic sequence downstream from the gRNA cleavage site, respectively, were employed to examine the on-target integration of donor sequence (Fig. S1 and S2). Some of the positive clones died after several rounds of subculture, the survived positive clones were selected and proceeded to downstream determinations.

(A)

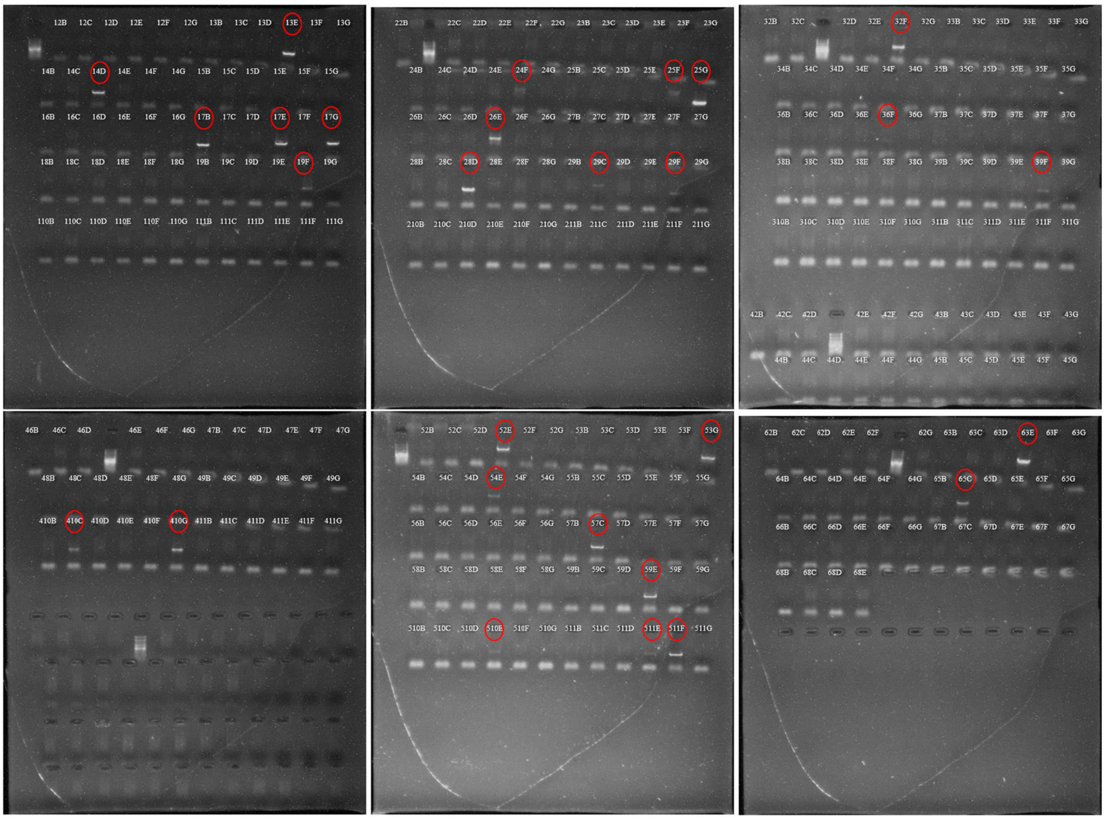

(B)

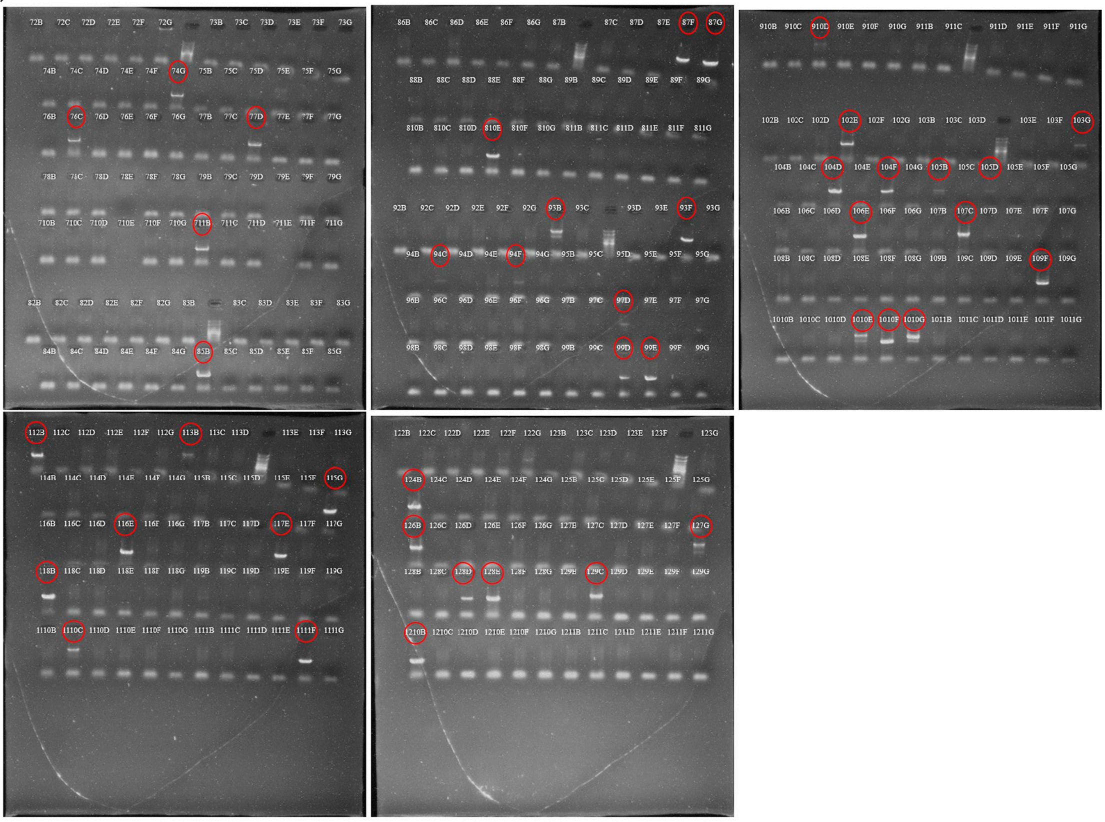

Figure S1. Screening of MDA-MB-231 knockout clones. The genomic DNA of MDA-MB-231 single-cell colonies transfected with gRNA1 (A) and gRNA2 (B) were PCR amplified using Puro5-fwd and SPAG5R3-rev primers to examine the on-target integration of donor sequence. A band of around 1071 bp was

expected in knockout clones. The cell colonies with on-target integration of donor sequence were circled in red colour.

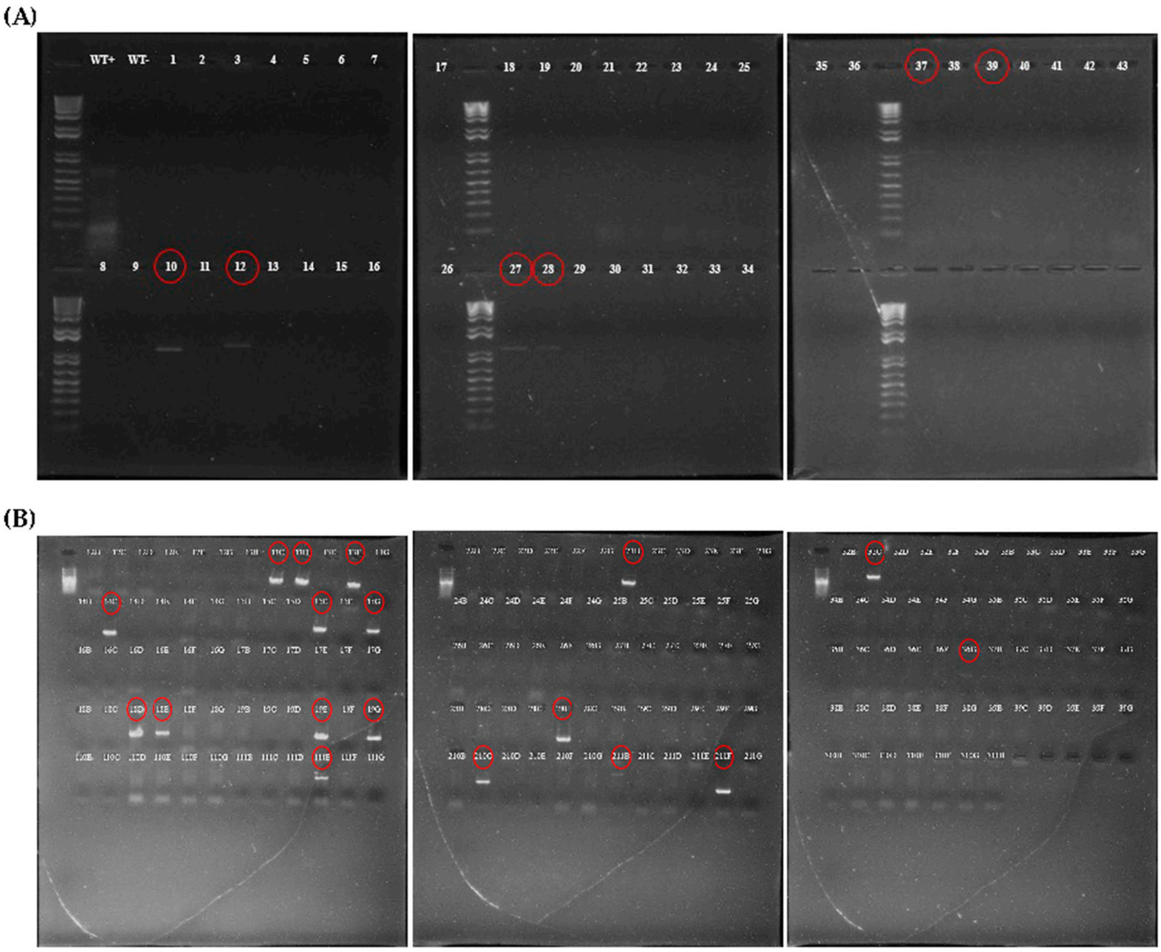

Figure S2. Screening of BT549 knockout clones. The genomic DNA of BT549 single-cell colonies transfected with gRNA1 (A) and gRNA2 (B) were PCR amplified using Puro5-fwd and SPAG5R3-rev primers to examine the on-target integration of donor sequence. A band of around 1071 bp was expected in knockout clones. The cell colonies with on-target integration of donor sequence were circled in red colour.

However, the above positive clones may be either homozygous or heterozygous knockout cells. Thus, a pair of primers (SPAG5F6-fwd and SPAG5R3-rev) flanking the cleavage site of gRNA were used to hybridize the *SPAG5* genomic sequence to examine whether both *SPAG5* alleles were with on-target integration of donor sequence. Unfortunately, all the positive clones were considered heterozygous knockout cells with a WT allele or an indel allele (Fig. S3).

(A)

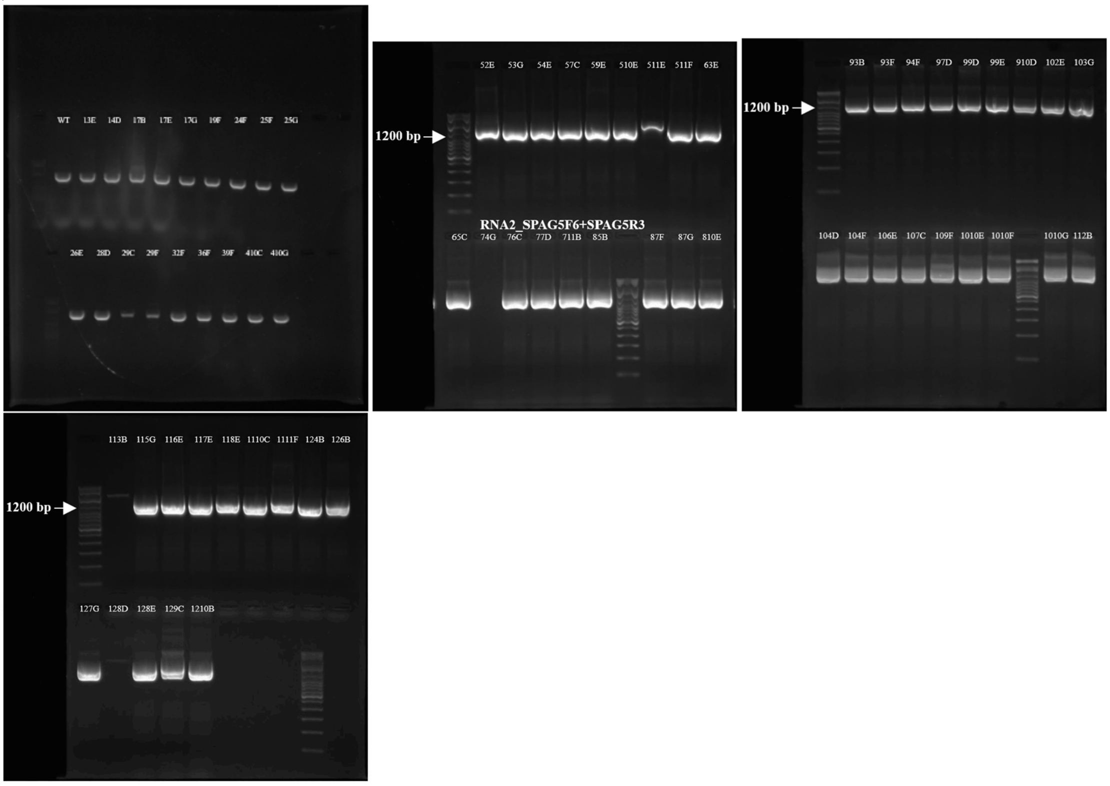

(B)

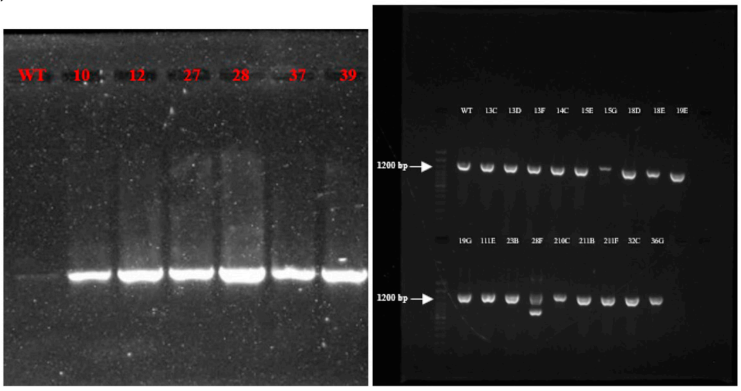

Figure S3. The genomic DNA of (A) MDA-MB-231 and (B) BT549 clones with on-target integration of donor sequence were PCR amplified using SPAG5F6-fwd and SPAG5R3-rev primers to examine the existence of WT/indel allele. A band of around 1385 bp was expected in the presence of the WT/indel allele. All the selected cell colonies with donor sequence were heterozygous knockout cells.

The allele without donor sequence in the above heterozygous clones might also be cleaved and repaired by the NHEJ pathway, which means that one allele was replaced by the donor sequence and the other allele was either intact or indel. The latter probably shifts the gRNA target sequence and interrupts *SPAG5* transcription considering the cutting site of gRNA1 is 3 bp downstream from *SPAG5* exon 1, and the cutting site of gRNA2 is just located in *SPAG5* exon 1. Thus, the WT or indel allele of the above heterozygous clones was PCR amplified and sequenced. DNA sequencing results demonstrated that 2 of the MDA-MB-231-gRNA1, 2 of MDA-MB-231-gRNA2, 1 of BT549-gRNA1 and 1 of BT549-gRNA2 clones were with an indel allele. Interestingly, all these clones had a "T" insertion at 3 bp downstream from *SPAG5* exon

1 in the indel allele, irrespective of the gRNA and cell lines. One of each gRNA1 and gRNA2 clones was selected and proceeded to downstream assays to eliminate single-cell variability. The sequencing results of the selected clones are shown in Fig. S4 and S5.

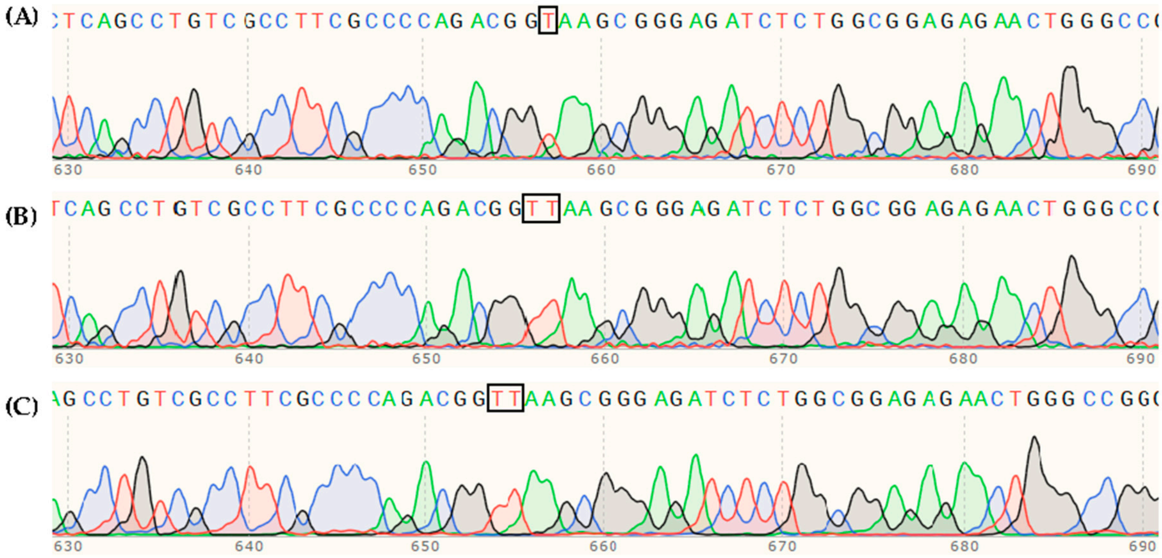

Figure S4. The target *SPAG5* gene sequence in MDA-MB-231 WT and knockout clones. (A) MDA-MB-231 WT cells; (B) M c1 cells; (C) M c2 cells.

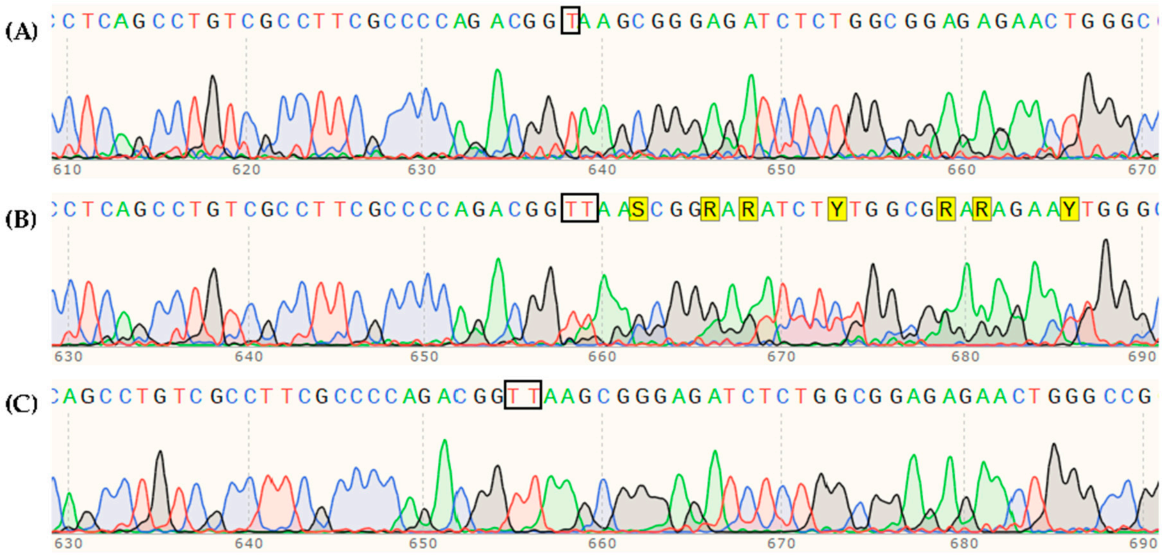

Figure S5. The target *SPAG5* gene sequence in BT549 WT and knockout clones. (A) BT549 WT cells; (B) B c1 cells; (C) B c2 cells.

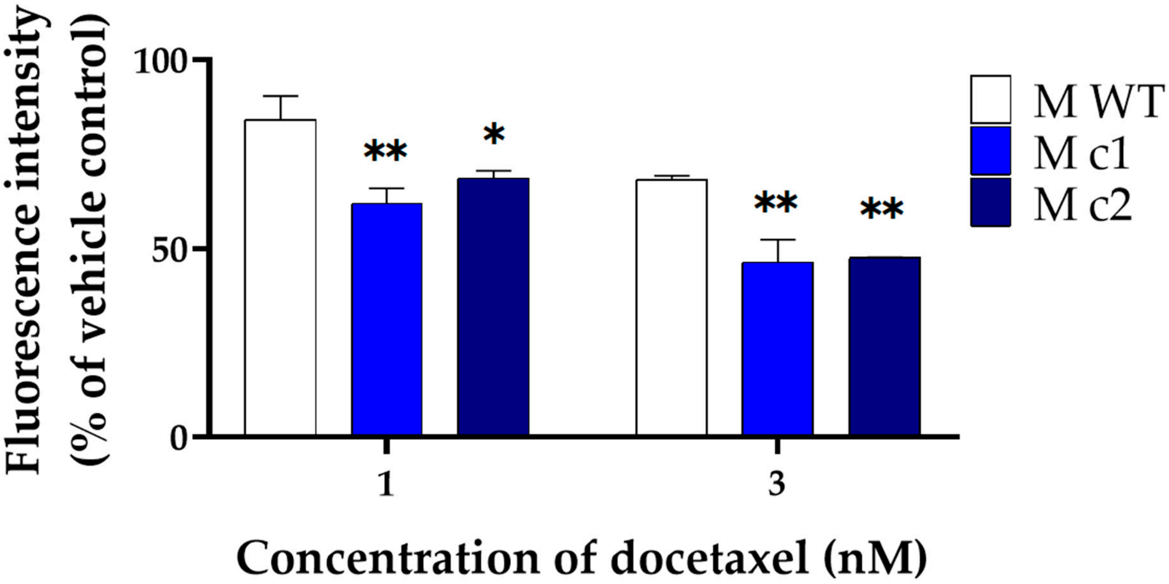

Figure S6. Docetaxel remarkably increased apoptosis rate in MDA-MB-231 knockout clones with downregulation of mitochondrial membrane potential. MDA-MB-231 knockout clones were treated with 1 nM and 3 nM docetaxel for 72 h. The mitochondrial membrane potential was analysed by TMRE-Mitochondrial Membrane Potential Assay Kit and flow cytometry. Data are presented as a mean percentage of vehicle control. The bar represents the mean and standard deviation of three independent experiments performed in triplicates. *, $P < 0.05$; **, $P < 0.01$ according to Sidak's post-hoc test that followed two-way ANOVA.
